# Microbial infections as potential risk factors for lung cancer: Investigating the role of human papillomavirus and chlamydia pneumoniae

**DOI:** 10.3934/publichealth.2023044

**Published:** 2023-08-03

**Authors:** Emmanuel Kwateng Drokow, Clement Yaw Effah, Clement Agboyibor, Jemima Twumwaah Budu, Francisca Arboh, Priscilla Akyaa Kyei-Baffour, Yao Xiao, Fan Zhang, Irene XY Wu

**Affiliations:** 1 Hunan Provinical Key Laboratory of Clinical Epidemiology, Central South University, Changsha 410083, Hunan, China; 2 Department of Epidemiology and Biostatistics, Xiangya School of Public Health, Central South University, Changsha 410083, Hunan, China; 3 General ICU, The First Affiliated Hospital of Zhengzhou University, Henan Key Laboratory of Critical Care Medicine, Zhengzhou Key Laboratory of Sepsis, Henan Engineering Research Center for Critical Care Medicine, Zhengzhou 450003, China; 4 School of Pharmaceutical Sciences, Zhengzhou University, Zhengzhou 450001, China; 5 School of Nursing, Zhengzhou University, 450001 Zhengzhou, China; 6 Department of Health Policy and Management, School of Management, Jiangsu University, 301 Xuefu Road, Zhenjiang, 212013 Jiangsu Province, China; 7 Department of General Surgery, Xiangya Hospital, Central South University, Changsha 410008, China; 8 University of Ghana Medical Center, Accra, Ghana; 9 National Clinical Research Center for Geriatric Disorders, Xiangya Hospital, Central South University, Changsha 410008, China; 10 Department of Gynecology, Xiangya Hospital, Central South University, Changsha, Hunan, China, 410008

**Keywords:** microbial infections, human papillomavirus, *chlamydia pneumoniae*, lung cancer, risk association

## Abstract

**Background:**

Lung cancer is the leading cause of cancer morbidity and mortality worldwide. Apart from tobacco smoke and dietary factors, microbial infections have been reported as the third leading cause of cancers globally. Deciphering the association between microbiome and lung cancer will provide potential biomarkers and novel insight in lung cancer progression. In this current study, we performed a meta-analysis to decipher the possible association between *C. pneumoniae* and human papillomavirus (HPV) and the risk of lung cancer.

**Methods:**

Literature search was conducted in most English and Chinese databases. Data were analyzed using CMA v.3.0 and RevMan v.5.3 software (Cochrane-Mantel-Haenszel method) by random-effects (DerSimonian and Laird) model.

**Results:**

The overall pooled estimates for HPV studies revealed that HPV infections in patients with lung cancer were significantly higher than those in the control group (*OR* = 2.33, 95% *CI* = 1.57–3.37, *p* < 0.001). Base on subgroup analysis, HPV infection rate was significantly higher in Asians (*OR* = 6.38, 95% *CI* = 2.33–17.46, *p* < 0.001), in tissues (*OR* = 5.04, 95% *CI* = 2.27–11.19, *p* < 0.001) and blood samples (*OR* = 1.40, 95% *CI* = 1.02–1.93, *p* = 0.04) of lung cancer patients but non-significantly lower in males (*OR* = 0.84, 95% *CI* = 0.57–1.22, *p* =0.35) and among lung cancer patients at clinical stage I-II (*OR* = 0.95, 95% *CI* = 0.61–1.49, *p* = 0.82). The overall pooled estimates from *C. pneumoniae* studies revealed that *C. pneumoniae* infection is a risk factor among lung cancer patients who are IgA seropositive (*OR* = 1.88, 95% *CI* = 1.30–2.70, *p* < 0.001) and IgG seropositive (*OR* = 1.50, 95% *CI* = 1.10–2.04, *p* = 0.010). All seronegative IgA (*OR* = 0.69, 95% *CI* = 0.42–1.16, *p* = 0.16) and IgG (*OR* = 0.66, 95% *CI* = 0.42–105, *p* = 0.08) titers are not associative risk factors to lung cancer.

**Conclusions:**

Immunoglobulin (IgA) and IgG seropositive titers of *C. pneumoniae* and lungs infected with HPV types 16 and 18 are potential risk factors associated with lung cancer.

## 1. Introduction

Lung cancer (LC) has been implicated as the leading cause of cancer morbidity and mortality (18.0% of the total cancer deaths) worldwide due to its poor survival rates post-diagnosis. According to the GLOBOCAN report, LC is the most diagnosed cancer type (11.4% of the total cases) [1]. In 2020, there was a global prediction of 2.2 million new LC cases. There are a lot of risk factors that are associated with LC. Based on epidemiological evidence, tobacco smoke, dietary factors, environmental pollution and genetic factors are some risk factors for LC. Evidence has suggested that microbial infections have also been associated with LC. According to de Xiong et al. [2], microbial infections are the third leading cause of cancer globally with 16.1% being the proportion of cancers associated with chronic pathogenic infections. The seemingly increase in research on the association between microbiome, LC and pulmonary diseases, have provided potential biomarkers and novel insight in disease progression [3]. Notable organisms that have been postulated to be associated with LC are Human papillomavirus (HPV) and *Chlamydia pneumoniae*.

Human papillomaviruses (HPV) are small DNA viruses belonging to the Papillomaviridae family. They are non-enveloped, circular, epitheliotropic, double-stranded DNA viruses which are widely known for its importance in cervical cancer and other anogenital and oropharyngeal cancers [4]. Based on the carcinogen ratings by the International Agency for Research on Cancer (IARC), several HPV types have been classified under Class I carcinogens (HPV16, 18, 31, 33, 35, 39, 45, 51, 52, 56, 58 and 59) while others are among the Class IIA and Class III groups [5]. The role of HPV in carcinogenesis depends on its ability to strongly attach to the skin and other mucosal tissues and also due to its ability to persist in infections and integrates its genetic material into the host genome. Furthermore, when various oncoproteins (E6 and E7) of HPV are expressed, they inactivate some tumor suppressors which accelerate the progression of tumorigenesis. When abrasions occur in epithelia tissues, the virus takes advantage to infect undifferentiated squamous epithelia cells that are beneath the epithelial tissue [6]. Because HPV has a high affinity to the squamous epithelium of bronchus and lung, it has been postulated that HPV is a possible related cause of lung neoplasms. A meta-analysis by Srinivasan et al., suggested that nearly 7% (95% *CI*: 4.7–10.6) of LC cases are associated with HPV16 and almost 6% (95% *CI*: 3.5–9.0) are associated with HPV18 [7]. Also, Ragin et al. (2014) reported that LC tissues are four more times likely to be HPV+ when compared to normal lung tissues [8].

*Chlamydia pneumoniae* is an obligate, intracellular, gram-negative bacillus with small and dense elementary body that causes pneumonia and other chronic respiratory tract infections and has been implicated to cause LC [9]. This organism is partially known to cause atherosclerotic cardiovascular disease and its detection in atherosclerotic plaque tissue [9],[10] as well as in specimens from lung, liver and spleen [11] indicates that it can persist chronically in the lung and other tissues after initial respiratory inoculation. Numerous studies evaluating the association between *C. pneumoniae* infection and LC risk have reported different risk estimates (ranges from 0.7 to 9.0) among individuals with seropositive titers [12]–[15]. There have been a reported 50% to 100% increased risks of LC among individuals with elevated IgA antibody titers in a series of prospective and retrospective studies. Kocazeybek, through a meta-analysis, reported an elevated level of IgA titers among LC patients which suggested that higher titers are higher predictors of LC risk than lower titers [13].

Regardless of the achievements made by different scholars in their effort to advance knowledge regarding association between human papillomavirus infection or *C. pneumoniae* infections and the risk of LC, there exists a knowledge gap that must be addressed due to the inconsistent results emanating from various studies. In this current study, we seek to answer the research question; what is the extent of the association between human papillomavirus (HPV) and *chlamydia pneumoniae* infections with the risk of developing lung cancer and how does this association vary across different populations by performed a meta-analysis to decipher the possible association between these organisms and the risk of lung cancer.

## 2. Methods and Materials

This research complied with the specified systematic reviews and meta-analyses (PRISMA) protocols and reporting requirements [16].

### 2.1. Searching procedure

Several English databases, including EMBASE, Cochrane Library, Web of Science, Medline/PubMed, Chinese Biomedical Literature Database (CBM) and Google scholar were searched for relevant literature. The search was done using the following search terms; “*Chlamydia pneumoniae*”, “*C. pneumoniae*”, “human papillomavirus”, “HPV”, “lung cancer”, “lung neoplasm”, “lung carcinoma and lung lesions”.

### 2.2. Exclusion and eligibility criteria

To be included, a study needed to have published findings on either human papillomavirus/LC or *C. pneumoniae*/LC. Prospective and retrospective cohort and case control studies were included. In vitro and animal studies, duplicate publications, conference proceedings, editorials were not included in the analysis.

### 2.3. Extraction of data

The extracted information from the final selected studies included sample size, period of study, location of study, publication year, study type, first author's name, age, gender, smoking status, clinical stage of cancer and cancer type.

### 2.4. Evaluation of Study Quality

The studies were evaluated by three authors using a quality rating scale. Discrepancies that arose during the review process were discussed and settled constructively. Studies were considered legitimate if they received a rating of 5 or above on the “Newcastle-Ottawa scale (NOS)” for non-randomized studies [17].

### 2.5. Statistical analysis

To estimate an association between the various microbial infections and LC risk, the Cochrane-Mantel-Haenszel method for dichotomous outcome variables and random-effects (DerSimonian-Laird) model were used [18]. The summary measure of association which provided weighted averages were presented as odds ratios (OR). Sensitivity and subgroup analyses were also conducted. Heterogeneity between-study was evaluated using the Q test. The leave-1-out sensitivity analyses were used to test the reliability of pooled evaluations and a study was deemed significant provided the pooled outcome excluding that study was higher than the 95% confidence interval (*CI*) of the total pooled estimate. Quantification of the degree of heterogeneity was done by *I^2^* statistics with the level of significance set at 0.05. Heterogeneity was achieved when *I^2^* was greater than 50%. Random effect model was adopted when heterogeneity was present; otherwise, a fixed effect model was used. Publication bias was evaluated using the funnel plots for visual analysis, and the Egger's test for quantitative significance. Data were analyzed using CMA v.3.0 and RevMan v.5.3 software by random-effects (DerSimonian and Laird) model.

## 3. Results

### 3.1. Systematic literature review and Study characteristics

**Figure 1.**
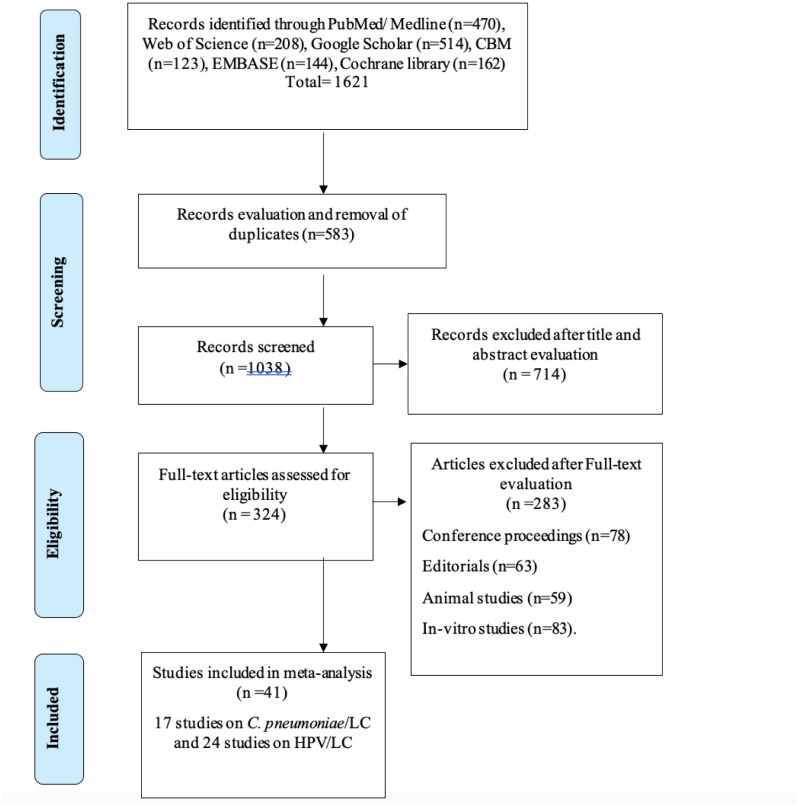
Prisma diagram of study procedure.

1621 studies were first gathered following a comprehensive search for literature (1080 studies on HPV/LC and 541 research on C. *pneumoniae*/LC). 583 articles were eliminated after data examination and screening for duplication. After reviewing the abstracts and study titles, seven hundred fourteen (714) articles were further excluded. 324 articles were subjected to the full-text evaluation, after which 283 articles were subsequently removed. The principal purposes for eliminating studies included in-vitro studies (83), animal studies (59), editorials (63) and conference proceedings (78). The eligibility requirement for this study were met by 41 studies; including 17 studies on C. *pneumoniae*/LC and 24 studies on HPV/LC. The Prisma diagram (Figure 1) shows a thorough description of the study's selection and screening process. Tables 1 and 2 describe the characteristics of included studies on *C. pneumoniae*/LC and HPV/LC respectively. Articles included in the analysis of the association between *C. pneumoniae* infection and LC risk involved 3365 cases and 3684 controls. Four studies (4) were nested case-control studies (1389 cases and 1696 controls) while thirteen studies (13) studies involving 1976 cases and 1988 controls were case control studies (Table S1). The analysis between HPV infection and the risk of LC were performed using a total of 5869 cases and 6394 from 24 studies (Table S2).

### 3.2. Quantitative synthesis

**Figure 2.**
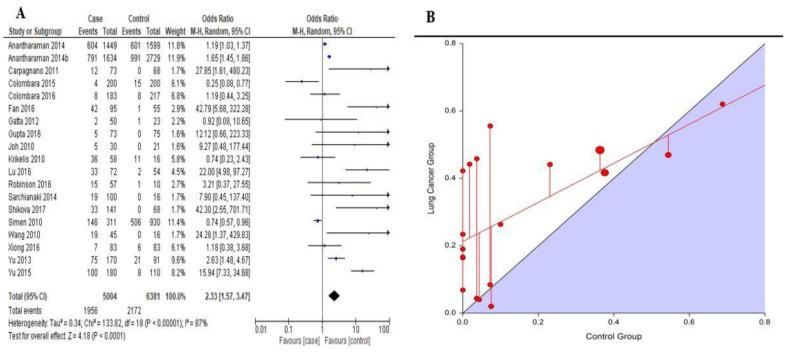
An association between HPV infection and the risk of lung cancer. (A) Forest plot for pooled HPV infection and the risk of lung cancer. (B) L'Abbe plot on the risk association between HPV infection and lung cancer.

**Figure 3.**
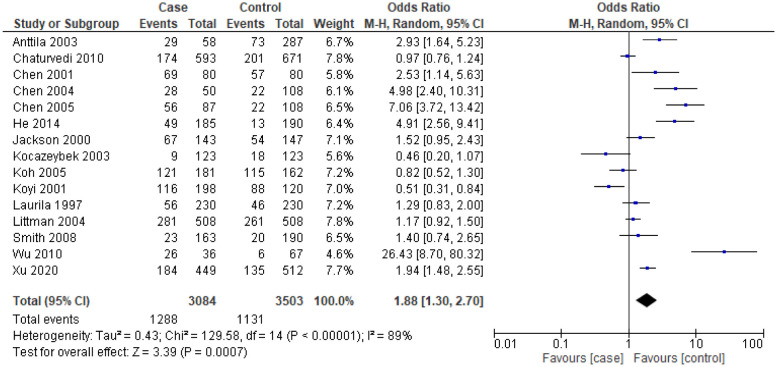
Forest plot for *C. pneumoniae* infection and the risk of lung cancer among patients with IgA seropositive titers.

The overall pooled estimates among 5004 cases and 6381 controls revealed that HPV infection was significantly higher among LC patients compared with their counterparts in the control group (*OR* = 2.33, 95%*CI*: 1.57–3.37) (Figure 2A) and this shows that HPV infection is a risk factor of LC. This was further confirmed through the L'Abbe plot (Figure 2B), where patients infected with HPV had a higher risk of developing lung cancer compared with those in the control group. L'Abbe plots the log risks on the scatterplot. The case-group log risk is on the y axis and the control-group log risk is on the x axis. The sizes of the plotted markers (circles) are proportional to the precision of the trials. Large circles represent more precise, larger studies, whereas small circles represent less precise, smaller studies. The log risks (or risks) in the two groups for the studies on the line are either the same or very similar. If a circle is above the reference line, the risk in the case group is higher than the risk in the control group for that study. Conversely, if a circle is below the line, the risk in the case group is lower than the risk in the control group. In this study, most of the trials are above the line, suggesting that the risk is higher in the case group. However, the studies demonstrating large differences between the groups are also smaller (less precise) studies. The HPV infection rates varied across continents and among the various cancer histological types. For squamous cell carcinoma (SCC) patients, the infection rates were 46.3%, 21.3% and 32.2% among Asians, Europeans and Americans respectively. For patients with adenocarcinoma (AC), the infection rates were 21.2 %, 9.5% and 10.5% among Asians, Europeans and Americans respectively (Figure 3). Base on subgroup analysis on continents, it was realized that HPV infection was a risk factor to LC, with Asians recording the highest risk (*OR* = 6.38, 95% *CI*: 2.33–17.46) followed by Americans (*OR* = 1.49, 95%*CI*: 0.14–15.46) and Europeans (*OR* = 1.29, 95% *CI*: 0.88–1.88) (Table 1). From the same table, it was noticed that HPV infection rates were significantly higher in tissues (*OR* = 5.04, 95% *CI*: 2.27–11.19) and blood (*OR* = 1.40, 95% *CI*: 1.02–1.93) samples of LC patients than the control group. Apart from LBMA (*OR* = 0.56, 95% *CI*: 0.12–2.59), all other detection techniques were significantly able to detect higher HPV rates in the LC group than in the control groups. From Table 2, it was realized that the risk of LC after HPV infection was higher in SCC patients (*OR* = 1.62, 95% *CI*: 0.62–4.29), smokers (*OR* = 1.41, 95% *CI*: 0.78–2.57) and patients with age less than 55years (*OR* = 1.09, 95% *CI*: 0.74–1.61). Patients infected with HPV type 16 are at a higher risk of developing lung cancer than those infected with type 18 (*OR* = 1.95, 95% *CI*: 1.00–3.79). On the contrary, HPV infection was not a risk factor in male LC patients (*OR* = 0.84, 95% *CI*: 0.57–1.22) and among LC cancer patients at stage I–II (*OR* = 0.95, 95% *CI*: 0.61–1.49).

**Table 1.** Subgroup analysis on HPV infections and the risk of lung cancer among cases and controls.

Subgroup	No. of studies	Case (n/N)	Control (n/N)	Test of association			Test of heterogeneity		
				*OR*	95% *CI*	*p*-valu*e*	*I^2^* (%)	*p-*value	*χ^2^*
**Continent**									
Asia	8	289/901	46/701	6.38	2.33–17.46	<0.001	82	<0.001	38.60
Europe	9	1660/4150	2110/5462	1.29	0.88–1.88	0.20	83	<0.001	45.91
America	3	24/287	16/231	1.49	0.14–15.46	0.74	76	0.02	8.17
**Sample type**									
Tissue	14	374/1466	50/664	5.04	2.27–11.19	<0.001	70	<0.001	42.95
Blood	2	1395/3083	1592/4328	1.40	1.02–1.93	0.04	91	<0.001	11.19
Serum	3	158/694	529/1347	0.66	0.34–1.27	0.22	55	0.11	4.42
Others	4	95/299	12/214	8.96	0.71–113.05	0.09	83	<0.001	17.40
**Detection technique**									
PCR	13	359/1409	49/654	5.24	2.25–12.22	<0.001	72	<0.001	42.94
Sequencing	2	17/103	0/89	18.04	2.38–136.71	0.005	<50	0.59	0.28
BMSM	2	1395/3083	1592/4328	1.40	1.02–1.93	0.04	91	<0.001	11.19
reverse blot hybridization	2	82/253	27/174	2.25	1.35–3.74	0.002	<50	0.22	1.52
LBMA	2	12/383	23/417	0.56	0.12–2.59	0.46	76	0.04	4.16
Others	5	297/733	529/1102	3.35	0.99–11.41	0.05	88	<0.001	34.68

Note: PCR: Polymerase chain reaction; LBMA: Multiplex liquid bead microarray antibody assay; BMSM: Bead-based multiplex serology method.

**Table 2.** Subgroup analysis on HPV infections and the risk of lung cancer among cases.

Subgroup	No. of studies	Test of association			Test of heterogeneity		
		*OR*	95% *CI*	*p-value*	*I^2^ (%)*	*p*-value	*χ^2^*
**Histological Type**							
SCC *vs*. AC	11	1.62	0.62–4.29	0.33	87	<0.001	74.92
**Smoking status**							
Smokers *vs*. non-smokers	11	1.41	0.78–2.57	0.26	64	0.002	27.96
**Gender**							
Male *vs*. Female	7	0.84	0.57–1.22	0.35	<50	0.88	2.41
**Clinical stage**							
I–II *vs*. III–IV	2	0.95	0.61–1.49	0.82	<50	0.41	0.69
**Age**							
<55 *vs*. ≥55	4	1.09	0.74–1.61	0.66	<50	0.51	2.33
**HPV type**							
16 *vs*. 18	4	1.95	1.00–3.79	0.05	59	0.06	7.36

Note: AC, Adenocarcinoma; SCC, Squamous cell carcinoma

**Figure 4.**
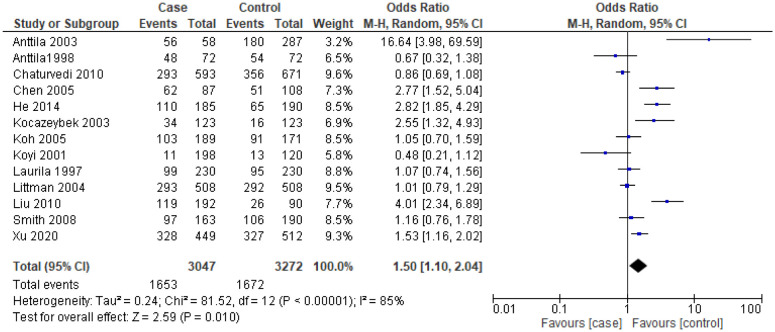
Forest plot for *C. pneumoniae* infection and the risk of lung cancer among patients with IgG seropositive titers.

The analysis between *C. pneumoniae* infection and the risk of LC revealed that the overall pooled estimates among patients with IgA seropositive (*OR* = 1.88, 95% *CI* = 1.30–2.70, *p* < 0.001, 3084 cases and 3503 controls from 15 studies) (Figure 3) and IgG seropositive titers (*OR* = 1.50, 95% *CI* = 1.10–2.04, *p* = 0.010, 3047 cases and 3272 controls from 13 studies) (Figure 4) were significantly higher in LC patients than in the control groups. With a subgroup analysis on the various type of studies, a significant higher titer of IgA in prospective studies (*OR* = 1.70, 95% *CI* = 1.08–2.69, *p* = 0.02, 1574 cases and 1886 controls from 5 studies) and retrospective studies (*OR* = 2.00, 95% *CI* = 1.13–3.54, *p* = 0.02, 1510 cases and 1617 controls from 10 studies) were reported. There was a border line significant on the seropositive titers of IgG among prospective studies that estimated the association between *C. pneumoniae* infections and the risk of LC (*OR* = 1.65, 95% *CI* = 1.01–2.68, *p* = 0.05, 1838 cases and 2048 controls from 7 studies) (Table 3). For nested case-control studies, *C. pneumoniae* seropositive titers for IgA and IgG in LC patients were non-significantly higher when compared with the control groups. On the contrary, case-control studies had significant higher seropositive titers of IgA and IgG in LC patients than in control groups. From Table 3 all seronegative IgA (*OR* = 0.69, 95% *CI* = 0.42–1.16, *p* = 0.16, 2113 cases and 2562 controls from 11 studies) and IgG (*OR* = 0.66, 95% *CI* = 0.42–105, *p* = 0.08, 1947 cases and 2307 controls from 8 studies) titers are not associative risk factors among LC patients.

### 3.3. Sensitivity analysis and Publication bias

The publication bias of the selected papers included in the study was evaluated using the Egger's test and Begg's funnel. The funnel plots obtained from the analysis conducted in this research are symmetrical, demonstrating that biased selection had no impact on our findings. Furthermore, the findings of the Egger test demonstrated that no substantial bias existed among our selected studies, since the *p-values* for all the pooled estimates were greater than 0.05 (*p* = 0.7201 for HPV/LC; *p* = 0.6210 for IgA seropositive titers of *C. pneumoniae* and *p* = 0.8905 for IgG seropositive titers of *C. pneumoniae*). The reliability or sensitivity of the pooled estimates analysis was then assessed using a leave-1-out sensitivity analysis. The statistical significance of the results did not change when a particular study was eliminated, demonstrating the reliability and validity of our study's conclusions.

## 4. Discussion

Certain microbial infections may be potential risk factors for the development and progression of certain cancers. Therefore, in other to facilitate the prevention and to improve the treatment strategies of cancer, there should be a holistic study to understand the mechanistic procedures of infection-mediated cancers. Because most single studies use small sample size and have less statistical power, meta-analysis which combines the results of these individual studies into a unified pooled effects can increase the sample size and the statistical power, help to detect modest risk differences among study groups and enable the evaluation of the association between *C. pneumoniae*/HPV infections and LC risk.

**Table 3.** Subgroup analysis on *C. pneumoniae* infections and the risk of lung cancer.

Subgroup	No. of studies	Case (n/N)	Control (n/N)	Test of association			Test of heterogeneity		
				*OR*	95% *CI*	*p-value*	*I^2^ (%)*	*p-value*	*χ^2^*
**Overall positive titers**									
IgA	15	1288/3084	1131/3503	1.88	1.30–2.70	<0.001	89	<0.001	129.58
IgG	13	1653/3047	1672/3272	1.50	1.10–2.04	0.010	85	<0.001	81.52
**Overall negative titers**									
IgA	11	1038/2113	1432/2562	0.69	0.42–1.16	0.16	92	<0.001	124.45
IgG	8	714/1947	907/2307	0.66	0.42–105	0.08	88	<0.001	56.23
**Type of study**									
Prospective									
IgA ≥ 16	5	589/1574	594/1886	1.70	1.08–2.69	0.02	87	<0.001	29.74
IgA < 16	5	855/1520	1162/1886	0.62	0.35–1.10	0.10	91	<0.001	45.77
IgG ≥ 32	7	1018/1838	1068/2048	1.65	1.01–2.68	0.05	90	<0.001	61.76
IgG < 32	5	594/1574	769/1886	0.53	0.29–0.99	0.05	90	<0.001	39.76
Retrospective									
IgA ≥ 16	10	699/1510	537/1617	2.00	1.13–3.54	0.02	90	<0.001	94.30
IgA < 16	6	183/539	325/676	0.43	0.11–1.67	0.22	95	<0.001	110.39
IgG ≥ 32	6	635/1209	604/1224	1.40	0.97–2.03	0.07	71	0.004	17.34
IgG < 32	3	120/373	138/421	0.89	0.36–2.20	0.80	88	<0.001	16.39
**Study design**									
Nested case-control									
IgA ≥ 16	4	540/1384	581/1696	1.39	0.94–2.05	0.10	81	0.001	15.43
IgA < 16	4	719/1389	985/1696	0.75	0.53–1.06	0.10	75	0.007	12.14
IgG ≥ 32	4	741/1389	923/1696	1.19	0.78–1.83	0.42	83	<0.001	17.26
IgG < 32	4	519/1389	644/1696	0.69	0.39–1.23	0.21	84	<0.001	18.47
Case-control									
IgA ≥ 16	11	748/1696	550/1807	2.17	1.26–3.76	0.005	90	<0.001	104.41
IgA < 16	7	319/720	502/866	0.39	0.12–1.25	0.11	95	<0.001	116.37
IgG ≥ 32	9	912/1656	749/1576	1.60	1.09–2.33	0.02	81	<0.001	42.82
IgG < 32	4	195/558	263/661	0.70	0.31–1.58	0.39	90	<0.001	30.30

Note: IgA, immunoglobulin A; IgG, immunoglobulin G.

**Figure 5.**
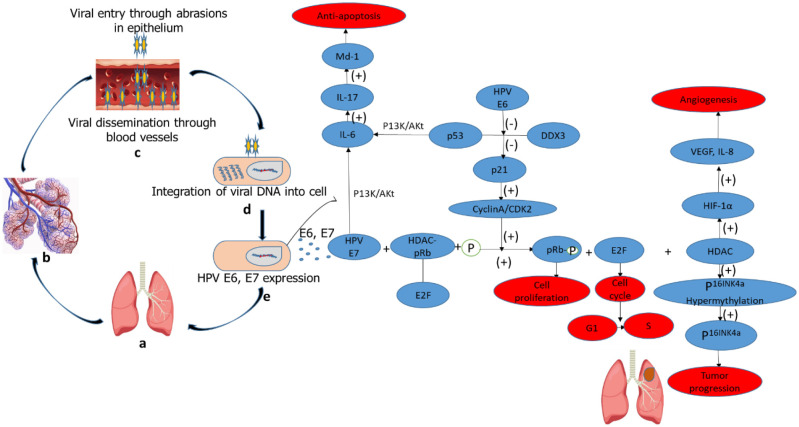
The mechanistic pathway of HPV infection which leads to lung cancer. **Note:** Alphabets **(a–b)** represent normal structures of the lung and alveoli. During abrasions on the lungs, DNA of HPV attaches and penetrates the epithelia cells of the lungs through blood vessels and pulmonary lunina **(c).** This DNA is recognized by pattern recognition receptors which leads to the integration of the viral DNA into the host cells **(d).** After integration, there is the expression of the viral oncoproteins E6 and E7 which play an important role in tumorigenesis **(e).** E6 oncoprotein suppresses the interaction between p53 and DDX3 which leads to the inactivation of p21. This then allows the phosphorylation of pRb by the free cyclinA/CDK2 complex which enhances cell proliferation, release of E2F transcription factor and also determines the cell cycle and G1/S transition. The HDAC/pRb/E2F complex is disrupted through its interaction with pRb when E7 oncoprotein is released. This then releases HDAC from the complex. HDAC causes 1. hypermethylation of p16INK4 which inhibits the expression of p16INK4 leading to tumor progression and 2. It can be induced by VEGF and IL-8 through HIF-1α to cause angiogenesis. The E6-inactivated p53 coupled with E7 oncoprotein can induce anti-apoptosis through up-regulation of Mcl-1 by the PI3K/Akt-(IL-6)-(IL-17) pathway. This figure was adopted and modified from Xiong et al. (2017).

According to a publication by Luo et al., oncoviruses, such as HPV accounts for about 12% of most cancers in humans [19]. When various oncoproteins (E6 and E7) of HPV are expressed, they inactivate some tumor suppressors or activate oncogenes which accelerate the progression of oncogenesis (Figure 5). Through various signal transduction pathways, oncoproteins E6 and E7 can facilitate lung cell proliferation, angiogenesis and cell immortalization by regulating the expression of multiple target genes and proteins such as p53, pRb, HIF-1α, VEGF, IL-6, IL-10, Mcl-1, Bcl-2, cIAP-2, EGFR, FHIT, hTERT, HER2, ALK, ROS1 and AhR [20]–[24]. Additionally, the upward regulation of PI3K and ERK pathways stimulated by Streptococcus and Veillonella, greatly increased in lung cancer compared to controls, suggests that the airway microbiome composition may contribute to the development of lung cancer by inducing signaling pathways associated with cancer development [25],[26].

In this study, there was a significant association of HPV infection and the risk of LC (*OR* = 2.33, 95% *CI* = 1.57–3.37, *p* < 0.001). This finding of this study confirms the results obtained by Xiong et al. and Maria et al. [27],[28] The infection rate varied from one geographical area to the other with Asia recording the highest rate of both SCC and AC histological types. The results from this study are in agreement with previous studies. According to a meta-analysis by Srinivasan et al., HPV prevalence in SCCs ranges from 0% to 48.1% and in ACs, from 0.0 to 44.4% [7]. In this study, the HPV prevalence ranged from 21.3% to 43.6% in SCC and 9.5% to 21.2% in AC. Also, an Iranian study detected HPV in 25% of SCCs and 21% of ACs [25],[29]. It has been purported that HPV infection is concomitant with certain histological subtypes of LC. A study by Yu et al. revealed that HPV infection rates were higher in SCC histological types (59.8%) than in AC histological types (17.5%) [30]. According to Fei et al., Chinese who lived in different areas showed different HPV infection prevalence [31]. From a research publication by Klein et al., the prevalence distribution pattern of HPV infection varies among Asians (35.7%), Americans (15%) and Europeans (17%) [32]. These observed variations in HPV prevalence among studies may be attributable to methodological issues [7],[32]. The geographic and environmental factors including education, economic and weather conditions may also account for the variable results.

HPV types 16 and 18 are the most predominant types that persist among LC patients. A meta-analysis by Zhai et al. has shown that the risk of developing SCC is strongly associated with lung infections that are caused by HPV16 and HPV18 [33]. The routes used by HPV during infection have been discussed elsewhere. Previous research had reported the detection of DNA sequence of HPV 16/18 in lung, blood and cervical smear [34], which suggests that HPV can be disseminated from the cervix to the lungs since the reproductive tract is the well-known niche of this organism. From this study, it was reported that HPV infection rates were significantly higher in tissues (*OR* = 5.04, 95% *CI* = 2.27–11.19, *p* < 0.001) and blood (*OR* = 1.40, 95% *CI* = 1.02–1.93, *p* = 0.04) samples of LC patients than in the control group. In other to achieve high sensitivity during HPV DNA detection, lung tissues have been recommended as the best sample type to use [35]. Also, apart from LBMA (*OR* = 0.56, 95% *CI* = 0.12–2.59, *p* = 0.46), all other detection techniques were significantly able to detect higher HPV rates in the LC group than in control groups. During the detection of HPV-Host DNA integration, PCR techniques are considered the ideal detection method. Also, the type of samples used during the detection process is of key importance. Serological samples and lung tissue samples whether fresh, paraffin embedded, formalin fixed or frozen can be utilized. For the serological sample, techniques such as BMSM or LBMA can be used [2]. However, one should have in mind that serological techniques have low sensitivity and specificity and that the number of HPVs that circulates in the blood are very limited. This makes lung tissue the best candidate for the detection of HPV oncoproteins. Serological methods can serve as alternative techniques during the detection of HPV proteins. However, there was a contradictory result from this study. In this meta-analysis, HPV proteins were significantly detected in both blood and tissue samples. In a previous meta-analysis by Xiong et al., a significant association was realized between HPV infection and LC risk (*OR* = 3.64; 95% *CI*: 2.60–5.08), with most of the studies used in this meta-analysis adopted PCR as the detection technique [2].

The epidemiology of LC may vary merely due to differences in environmental conditions, gene polymorphism and other factors such as smoking, HPV prevalence and sexual behavior which may contribute to higher HPV infection rates in LC patients. A synergistic relationship has been reported between microbial infections and cigarette smoking. Smoking can decrease the population of Langhans cell; an antigen presenting cell in epithelial tissue, which can lead to immune deficiency. Also, smoking weakens the active immune response system by releasing interleukin (IL)-4. Interleukin (IL)-4 thereby causes the release of T-helper (Th2) cells. These cells have an ineffective microbial clearing power in the lungs which consequently increases the microbial colonization in the lungs of patients that smoke [36],[37]. From this meta-analysis, HPV infection rates were non-significantly higher among smokers (*OR* = 1.41, 95% *CI* = 0.78–2.57, *p* = 0.26) when compared with non-smokers. Results from IARC (2007) revealed that HPV high-risk types were detection at a relatively high frequency among LC patients with AC histological type. Despite the fact that cigarette smoking has being reported as the primary risk factor for LC, nearly 15%–35% of non-small cell lung cancers (NSCLC) occur in non-smokers [38].

Littman et al, through an epidemiological study iterated that *C. pneumoniae* infection is a potential risk factor for the progression of lung cell carcinoma [39]. *C. pneumoniae* may down-regulate apoptosis during chronic infections by increasing the release of IL-10 [40]. According to reports from earlier studies, *C. pneumoniae* co-associate with other factors to facilitate carcinogenesis [13],[39],[41]. Moreover, some epidemiological evidence has suggested that infection in the respiratory tract is initiated by the infection of monocytes [42],[43]. In an in vitro study, Redecke et al. reported that alveolar macrophages from healthy persons release TNF-α, IL-1β, IL8 and superoxide oxygen radicals which play a vital role in the lung and during DNA damage [44]. *C. pneumoniae* is known as an effective inducer of TNF-α, IL-1β and IL-6 in host monocytic cells and has the potential to induce carcinogenesis. *C. pneumoniae*, which proliferates in the monocytes and macrophages, initiates pathogenesis. Pathogenesis involves a series of processes which includes adaptive and non-adaptive immune response to the antigenic material, infection of new monocytes, endothelial cells and macrophages in the intima and smooth muscle cells in the media layer which activates the release of cytokines and acute-phase proteins to produce chronic inflammation. This inflammation may result in cell injury and in an attempt to repair the injury, cell division occurs uncontrollably and this increases the risk of mutation which may lead to cancer [11].

Despite the above mechanistic evidence of *C. pneumoniae* in LC, a series of research conducted to confirm the possible association between *C. pneumoniae* infection and the risk of LC has yielded inconsistent results [45]. According to Zhan et al., C. pneumoniae infection raises the likelihood of developing lung cancer. However, a greater titre may be a better indicator of that risk [46]. Similar findings were reported by other studies [47],[48]. Xu et al., concluded that C. *pneumoniae* infection may cause primary lung cancer in the Chinese Han populace [49].

In this study, it was found that the titers of *C. pneumoniae* antibodies were elevated in LC patients as compared with their control counterparts. This was in agreement with Littman et al. who iterated that patients with higher titers of IgA are at a 10-fold higher risk of SCC and AC of the lung [39]. Definition of chronic *C. pneumoniae* infection varies among studies with some using combination of antibody titers [13],[41] while others use one or more antibodies of IgG or IgA alone [11],[50],[51]. Although it is biologically unclear how higher titers may be related to severity or chronicity of infection, the significantly elevated levels of IgA titers ≥ 16 and IgG ≥ 32 (higher ORs in cases than in controls) realized in this study suggests that higher titers may be a better predictor of lung cancer risk than lower antibody titers.

In a retrospective study, baseline exposure be it suspected risk or protection factor, is assessed at some point in the past through historical records that is established at the start of the study. In a prospective study, baseline exposure is assessed at the beginning of the study and is followed into the future. It minimizes any selection bias and generally includes better adjustment of potential confounding factors. To investigate whether there was heterogeneity among the type of studies, subgroup analysis was performed and the results iterated that there was a significant heterogeneity among both retrospective and prospective studies. In this section of this meta-analysis, subgroup analyses were not performed on age, gender and smoking status. These variables are potentially important confounders and are strongly associated with LC and elevated levels of IgA and IgG titers of *C. pneumoniae* [52],[53]. Inadequate control for these factors could result in a bias away from the null.

*C. pneumoniae* is a bacterium that can cause respiratory tract infections, including pneumonia. On the other hand, human papillomavirus (HPV) types 16 and 18 are known to be high-risk types of HPV associated with various types of cancer, including lung cancer [54]–[57]. Although the exact mechanisms are not fully understood, there are several ways in which the immunoglobulin (IgA and IgG) seropositive titers of *C. pneumoniae* and the presence of HPV types 16 and 18 in the lungs may be potential risk factors associated with lung cancer [58]–[60].

Both *C. pneumoniae* and HPV infections can cause chronic inflammation in the lungs. Chronic inflammation creates an environment that promotes the growth and progression of cancer cells. It can lead to DNA damage, cellular mutations and an impaired immune response, all of which can contribute to the development of cancer [61],[62]. Secondly, IgA and IgG antibodies are part of the immune response against infections. In the case of *C. pneumoniae*, increased IgA and IgG seropositive titers suggest an ongoing or past infection. Elevated antibody levels may indicate a prolonged immune response and persistent infection [61]–[63]. Similarly, HPV infection triggers an immune response, leading to the production of antibodies. Dysregulation of the immune response, either due to prolonged or inadequate response, may contribute to the progression of HPV-associated lung cancer [61]–[64].

Furthermore, evidence suggest that *C. pneumoniae* and HPV infections may have a synergistic effect on promoting lung cancer development [65],[66]. Both infections can independently induce cellular changes and genetic instability, increasing the susceptibility to malignant transformation [67]–[69]. The combination of these infections may lead to more severe and long-lasting inflammation and immune dysregulation, further promoting the development of lung cancer [67]–[69]. Additionally, *C. pneumoniae* and HPV infections have been associated with DNA damage and genomic instability. *C. pneumoniae* can directly infect lung epithelial cells, leading to DNA damage [68],[69]. On the other hand, HPV types 16 and 18 have viral oncogenes that can interfere with cellular DNA repair mechanisms, increasing the risk of genetic mutations and chromosomal abnormalities. These genetic alterations can contribute to the initiation and progression of lung cancer.

### 4.1. Strengths and limitations

The use of two important organisms to demonstrate risk associations and the implementation of a very comprehensive searching approach are the strengths of this study. Sensitivity analysis, which systematically omitted one test at a time, showed that the results of this study were consistent, signifying the study's reliability. The inclusion of publications from America, Asia and Europe increased the generalizability and reliability of our findings. This study has some limitations, including the inability to do subgroup analyses for *C. pneumoniae*/LC risk based on smoking status, age, cancer histological types or gender due to a lack of primary study data. Additionally, some of the studies included were only fair quality. Most of the study adopted a retrospective approach which makes them potentially biased towards certain groups. This study is only partially able to show a causal association because it was impossible to present a targeted account taking into account all confounders.

## 5. Conclusion

In conclusion, the available evidence suggests that certain microbial infections, specifically HPV and *C. pneumoniae*, may play a role as potential risk factors in the development and progression of lung cancer (LC). The oncogenic properties of HPV, particularly types 16 and 18, have been found to be associated with an increased risk of LC. The prevalence of HPV infection varies across geographical regions and histological subtypes of LC. Detecting HPV DNA in lung tissues using PCR techniques has been recommended for high sensitivity. On the other hand, chronic *C. pneumoniae* infection has been implicated in LC by down-regulating apoptosis and inducing chronic inflammation, which can lead to cell injury and an elevated risk of mutation. However, it is important to note that the association between these infections and LC risk is influenced by various factors such as environmental conditions, gene polymorphism, smoking and sexual behavior.

Governments should develop policies to promote regular screening programs for lung cancer, including targeted screening for high-risk populations such as smokers and individuals with a history of viral infections like HPV. These programs can help identify cases at an early stage when treatment options are more effective. Health policies should focus on comprehensive prevention strategies that address both viral infections and risk factors like smoking. This can involve public awareness campaigns, education programs and initiatives promoting healthy lifestyle choices including smoking cessation programs and vaccination against HPV. Health policies should emphasize non-pharmaceutical measures to control the spread of HPV and associated cancers. This can include promoting safe sexual practices such as condom use and reducing the number of sexual partners. Additionally, policies should encourage regular health check-ups and HPV testing to detect infections early and provide appropriate interventions. Governments should allocate resources and funding to support research on the relationship between viral agents, particularly HPV and lung cancer. Collaboration between researchers, healthcare institutions and policymakers can facilitate the development of targeted therapies and innovative treatment approaches to combat HPV-associated lung cancers.

## Use of AI tools declaration

The authors declare they have not used Artificial Intelligence (AI) tools in the creation of this article.
