# Supplementary material for: Microbial infections as potential risk factors for lung cancer: Investigating the role of human papillomavirus and chlamydia pneumoniae
Source: AIMS Public Health. 2023 Aug 3;10(3):627–46. doi: 10.3934/publichealth.2023044 (PMC10567973; doi:10.3934/publichealth.2023044)
Supplement: Supplementary file 1 [file publichealth-10-03-044-s001.pdf]

Public Health

AIMS Public Health, 10(3): 627–646.
DOI: 10.3934/publichealth.2023044
Received: 10 April 2023
Revised: 22 June 2023
Accepted: 03 July 2023
Published: 03 August 2023

http://www.aimspress.com/journal/aimsph

Research article

Microbial infections as potential risk factors for lung cancer: Investigating the role of human papillomavirus and chlamydia pneumoniae

Emmanuel Kwateng Drokow[1,2,*,†], Clement Yaw Effah[3,†], Clement Agboyibor[4], Jemima Twumwaah Budu[5], Francisca Arboh[6], Priscilla Akyaa Kyei-Baffour[7], Yao Xiao[8,9], Fan Zhang[10,*], and Irene XY Wu[1,2,*]

[1] Hunan Provinical Key Laboratory of Clinical Epidemiology, Central South University, Changsha 410083, Hunan, China
[2] Department of Epidemiology and Biostatistics, Xiangya School of Public Health, Central South University, Changsha 410083, Hunan, China
[3] General ICU, The First Affiliated Hospital of Zhengzhou University, Henan Key Laboratory of Critical Care Medicine, Zhengzhou Key Laboratory of Sepsis, Henan Engineering Research Center for Critical Care Medicine, Zhengzhou 450003, China
[4] School of Pharmaceutical Sciences, Zhengzhou University, Zhengzhou 450001, China
[5] School of Nursing, Zhengzhou University, 450001 Zhengzhou, China
[6] Department of Health Policy and Management, School of Management, Jiangsu University, 301 Xuefu Road, Zhenjiang, 212013 Jiangsu Province, China
[7] Department of General Surgery, Xiangya Hospital, Central South University, Changsha 410008, China
[8] University of Ghana Medical Center, Accra, Ghana
[9] National Clinical Research Center for Geriatric Disorders, Xiangya Hospital, Central South University, Changsha 410008, China
[10] Department of Gynecology, Xiangya Hospital, Central South University, Changsha, Hunan, China, 410008

* **Correspondence:** Emmanuel Kwateng Drokow: Email: kwateng251@gmail.com; kwateng@csu.edu.cn. Fan Zhang: Email: kellyzfhuan@163.com. Irene XY Wu: Email: irenexywu@csu.edu.cn.

† These two authors contributed equally.

Supplement I. Characteristics of studies included for the association between C. pneumoniae infection and the risk of lung cancer.

Author (year)	Study design	Sample size (Case: control)	Age (Case: control	Case (n/N)				Control (n/N)			
				IgA (<16)	IgA (≥16)	IgG (<32)	IgG (≥32)	IgA (<16)	IgA (≥16)	IgG (<32)	IgG (≥32)
Laurila (1997)	Nest case-control, Pros.	230:230	60.3: 60.3	44/230	56/230	2/230	99/230	54/230	46/230	5/230	95/230
Anttila (1998)	case–control, Pros	72:72	---	---	---	---	48/72	---	---	---	54/72
Smith (2008)	case–control, Retro	163:190	---	57/163	23/163	31/163	97/163	62/190	20/190	35/190	106/190
Jackson (2000)	case–control, Retro	143: 147	---	---	67/143	---	---	---	54/147	---	---
Koh (2005)	case–control, Retro	200: 181	65.8: 63.6	---	121/181	---	103/189	---	115/162	---	91/171
Koyi (2001)	case–control, Retro	198:120	---	---	116/198	---	11/198	---	88/120	---	13/120
Kocazeybek (2003)	case–control, Retro	123:123	55.0: 55.0	52/123	9/123	64/123	34/123	7/123	18/123	46/123	16/123
Wu (2010)	case–control, Retro	36:67	---	10/36	26/36	---	---	61/67	6/67	---	---
Chen (2005)	case–control, Retro	87:108	50.9 ± 11: 48.1 ± 10.1	31/87	56/87	25/87	62/87	86/108	22/108	57/108	51/108

Continued on next page

Author (year)	Study design	Sample size (Case: control)	Age (Case: control	Case (n/N)				Control (n/N)			
				IgA (<16)	IgA (≥16)	IgG (<32)	IgG (≥32)	IgA (<16)	IgA (≥16)	IgG (<32)	IgG (≥32)
Anttila (2003)	Nested case–control, Pros	58:287	32.0: 41.0	29/58	29/58	2/58	56/58	214/287	73/287	107/287	180/287
Chen (2004)	case–control, Retro	50:108	NA: 40.8 ± 8.5	22/50	28/50	---	---	86/108	22/108	---	---
Chaturvedi (2010)	nest case-control, Pros	593: 671	---	419/593	174/593	300/593	293/593	470/671	201/671	315/671	356/671
Xu (2020)	case–control, Retro	449: 512	---	---	184/449	---	328/449	---	135/512	---	327/512
Liu (2010)	case-control, Pros	192: 90	54.6 ± 10.4:53.6 ± 9.4	---	---	---	119/192	---	---	---	26/90
Littman (2004)	nest case-control, Pros	508: 508	59: 59	227/508	281/508	215/508	293/508	247/508	261/508	217/508	292/508
Chen (2001)	case–control, Retro	80: 80	58 ± 17: 57 ± 19	11/80	69/80	---	---	23/80	57/80	---	---
Fei (2014)	case–control, Pros	185:190	58.57 ± 9.49: 57.96 ± 9.28	136/185	49/185	75/185	110/185	177/190	13/190	125/190	65/190

Supplement II. Characteristics of studies included for the association between HPV infection and the risk of lung cancer.

Author (Year)	Country	HPV types	Sample type	Detection technique	Case (n/N)	Control (n/N)
					HPV (+)	HPV (+)
Colombara (2016)	China	6, 11, 16, 18, 31, 33, 52, 58	Serum	LBMA	8/183	8/217
Xiong (2016)	China	21 types	tissue	PCR, reverse blot hybridization	7/83	6/83
Fan (2016)	China	16	PE	ICC	42/95	1/55
Robinson (2016)	USA	28 types	tissue	microarray, oncovirus panel, genotyping PCR	15/57	1/10
Gupta (2016)	India	16, 18, 31, 33, 45	FNAC, tissue	PCR	5/73	0/75
Lu (2016)	China	16, 18	tissue	PCR	33/72	2/54
Yu (2015)	China	L1, 16, 18	tissue	PCR	100/180	8/110
Colombara (2015)	USA	6, 11, 16, 18, 31, 33, 52, 58	Serum	LBMA	4/200	15/200
Sarchianaki (2014)	Greece	37 types	tissue	PCR, genotyping	19/100	0/16
Anantharaman (2014)	10 European countries	6, 11, 16, 18, 31	Blood	BMSM	604/1449	601/1599
Sagerup (2014)	Norway	15 types	tissue	PCR	13/334	0/13
Anantharaman (2013)	7 European countries	6, 11, 16, 18, 31	Blood	BMSM	791/1634	991/2729
Joh (2010)	USA	NA	tissue	PCR, sequencing	5/30	0/21
Gatta (2012)	Italy	16, 18, 33, 35, 52, 58	tissue	PCR	2/50	1/23

Continued on next page

Author (Year)	Country	HPV types	Sample type	Detection technique	Case (n/N)	Control (n/N)
					HPV (+)	HPV (+)
Yu (2013)	China	25 types	tissue	PCR, reverse blot hybridization, SB	75/170	21/91
Wang (2010)	China	16, 18	tissue	PCR	19/45	0/16
Krikelis (2010)	Greece	16	tissue, BW	PCR	36/58	11/16
Shikova (2017)	Bulgaria	16, 18	Tissue	consensus PCR, TS PCR	33/141	0/68
Kato (2012)	Japan	16, 18	Tissue	PCR	7/42	NA
Koshiol (2011)	Italy	16. 18	Tissue	PCR	0/388	NA
Goto (2011)	Japan	16/18	Tissue	PCR, ISH	1/44	NA
Carpagnano (2011)	Italy	16, 30, 31, 39	Tissue, EBC, bronchial brushing	PCR, Pyrosequencing	12/73	0/68
Baba (2010)	Japan	6, 16, 18, 33	Tissue	PCR	11/57	NA
Simen (2010)	Finland	16, 18	Serum	ELISA	146/311	506/930

AIMS Press

© 2023 the Author(s), licensee AIMS Press. This is an open access article distributed under the terms of the Creative Commons Attribution License (http://creativecommons.org/licenses/by/4.0)
